# The image dataset of Indian coins: A machine learning approach for Indian currency

**DOI:** 10.1016/j.dib.2024.110098

**Published:** 2024-01-24

**Authors:** Yogesh Suryawanshi, Vidula Meshram, Kailas Patil, Michael Testani, Prawit Chumchu, Anmol Sharma

**Affiliations:** a Vishwakarma University, India; b Vishwakarma Institute of Information Technology, India; c Binghamton University, USA; d Kasetsart University, Sriracha, Thailand

**Keywords:** Indian coinage, Denomination, Classification, Modern currency, Coin verification

## Abstract

In an increasingly digital world, the significance of creating a Comprehensive Image Dataset of Contemporary Indian Coins (CIDCIC) cannot be overstated. This research presents a dataset comprising 6,672 images of 53 different classes of Indian coins, including denominations of 25 Paisa, 50 Paisa, 1 Rupee, 2 Rupee, 5 Rupee, 10 Rupee, and 20 Rupee. The images of coins with various shapes and sizes are taken from obverse and reverse sides in various environments and different backgrounds. The core significance of this dataset unfolds in its potential to offer invaluable assistance to visually impaired individuals as they navigate their daily financial transactions. The dataset is a significant contribution to the domains of computer vision, artificial intelligence, and machine learning, specifically addressing the challenges related to coin detection, recognition, and monetary system integrity. These technologies can empower visually impaired individuals to independently and accurately recognize and distinguish between various coin denominations, thereby enhancing their participation in the financial realm. The dataset addresses limitations in existing dataset of having limited size, and scope. It addresses the limitations associated to the limited number of coins and the lack of diversity in images, encompassing various angles, environments, backgrounds, and directions of coins. The dataset provides a broader and more up-to-date representation of contemporary Indian coins.

Specifications Table

**Table:** 

Subject	Finance and Banking, Computer Science
Specific subject area	Asset Pricing, Computer Science Applications, Applied Machine Learning
Data format	Raw
Type of data	Image
Data collection	The images of coins of various shapes and sizes of denomination 25 Paisa, 50 Paisa, 1 Rupee, 2 Rupee, 5 Rupee, 10 Rupee, and 20 Rupee are captured using mobile phone's rear camera. The images are taken in different backgrounds, lighting conditions, and angles, capturing both the obverse and reverse sides of the coin. The image dimensions are 575 × 768 and are in jpg format. As per denominations the coin images are segregated in respective folders.
Data source location	Vishwakarma University, Laxmi Nagar, Kondhwa Budruk, Pune – 411 048. Maharashtra, India. Latitude and longitude: 18.4603°N, 73.8836°E
Data accessibility	Repository name: Image Dataset of Indian Coin Data identification number: 10.17632/txn6vz28g9.2 Direct URL to data: https://data.mendeley.com/datasets/txn6vz28g9/2

## 1 Value of the Data

• This dataset comprises 6672 high-quality images (25 Paisa Coin, 50 Paisa Coin, 1 Rupee Coin, 2 Rupee, 5 Rupee Coin, 10 Rupee Coin, and 20 Rupees Coin) of commonly used contemporary Indian coins, providing a comprehensive collection.
• In dataset the images captured from both sides of the coins, with varied angles, backgrounds, and lighting conditions for robust dataset coverage.
• The dataset can be a valuable resource for researchers engaged in the field of employing machine learning techniques to distinguish authentic and counterfeit coins, and also for recognizing between different denomination values.
• Leveraging technology for coin classification can significantly improve the ability of visually impaired individuals to identify and categorize coins, thereby promoting greater accessibility and independence in financial transactions.
• The dataset provides a visual timeline of coinage evolution, offering insights into economic trends, political changes, and cultural influences over time. Culturally, the dataset contributes to preserving the visual heritage of Indian currency, capturing the nuances of design and symbolism intrinsic to each coin. The dataset is crucial for the development and refinement of computer vision and machine learning algorithms for coin classification, thus it holds immense value across diverse domains, notably as it offers potential applications in numismatic studies, historical research, cultural preservation, and technological advancements.

## 2 Background

This research presents a dataset comprising 6672 images of 53 different classes of Indian coins, including denominations of 25 Paisa, 50 Paisa, 1 Rupee, 2 Rupees, 5 Rupees, 10 Rupees, and 20 Rupees. The images of coins with various shapes and sizes are taken from obverse and reverse sides in different various environments and different backgrounds. The core significance of this dataset unfolds in its potential to offer invaluable assistance to visually impaired individuals as they navigate their daily financial transactions. The dataset is a significant contribution to the domains of computer vision, artificial intelligence, and machine learning, specifically addressing the challenges related to coin detection, recognition, and monetary system integrity. These technologies can empower visually impaired individuals to independently and accurately recognize and distinguish between various coin denominations, thereby enhancing their participation in the financial realm. The dataset provides a broader and more up-to-date representation of contemporary Indian coins.

## 3 Data Description

Indian coins have a rich history dating back thousands of years. They showcase the cultural, political, and economic evolution of the Indian subcontinent. From ancient punch-marked coins to the intricately designed coins of empires like the Mauryas and Guptas, each piece tells a unique story. Today, the Reserve Bank of India issues a range of coins denominated in paisa and rupees. These coins used in day to day life for various transactions. The rupee is the official currency of India, serving as the primary unit of monetary exchange. Symbolized by ₹, the rupee is subdivided into smaller units known as paisa, with 100 paisa equaling 1 rupee. While the term ``paisa'' continues to be used to denote fractional amounts, coins in denominations of 1 paisa and 2 paisa are no longer in circulation due to their nominal value. The coin series in active use includes 25 paisa, 50 paisa, 1 rupee, 5 rupee, 10 rupee and 20 rupee, while banknotes cover higher denominations. The rupee is a vital component of India's financial system and plays a central role in daily economic transactions.

Denomination classification and identification is an vital task for visually impaired people to do the financial transactions independently [[1], [2]], as well automatic recognition of coins is a significant task for vending machines, several equipment related to banking [3]. Several deep learning and machine learning algorithms are employed for classification and recognition of coins [[4], [5]]. To avoid substantial societal loss and damages identification of fake coins is a crucial task [[6], [7]]. As per [[6], [8], [9]] dataset plays an important role for machine learning algorithms to perform well, also dataset of Indian coins is important aid for historical and numismatics study [10].

The existing coin datasets do not contain all modern day Indian coins and are small in size. This comprehensive and contemporary coin image dataset is created in order to facilitate the development and progress of computer vision, machine learning, and artificial intelligence technologies specifically geared towards the recognition, categorization, and authenticity of Indian coins, an ``Image Dataset of Indian Coins'' has been created.

**Fig. 1:**
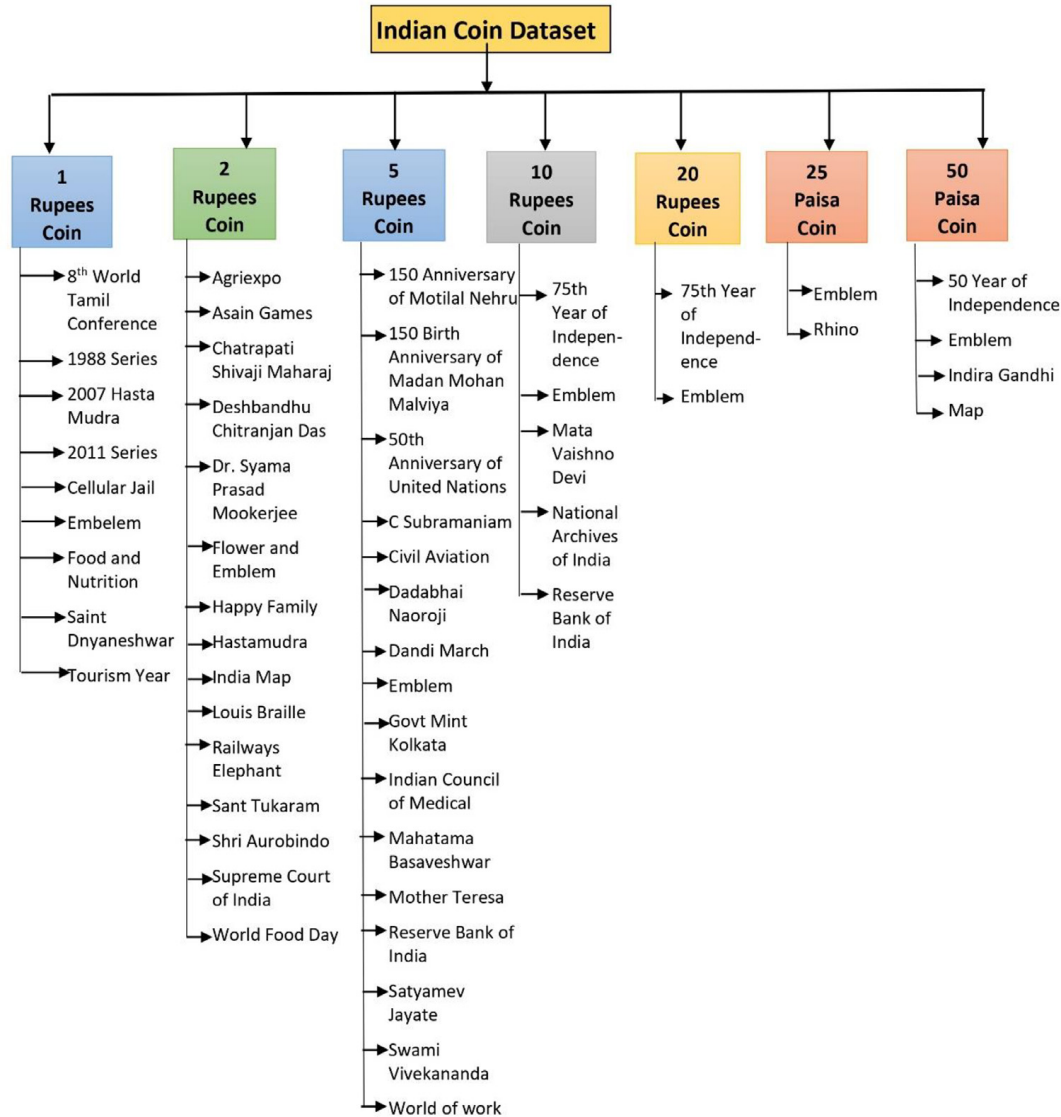
Detail directory structure of Indian coin dataset.

The dataset ``Image Dataset of Indian Coins” [11] is comprehensive and contemporary, consisting images of Indian coins with the denominations of 25 Paisa, 50 Paisa, 1 Rupee, 2 Rupee, 5 Rupee, 10 Rupee, and 20 Rupee. The dataset comprises folders dedicated to different coin denominations: the 1 Rupee coin folder encompasses 9 subfolders, totaling 779 images; the 2 Rupee coin folder consists of 15 subfolders, amounting to 2042 images; the 5 Rupee coin folder includes 16 subfolders, summing up to 1918 images; the 10 Rupee coin folder is organized into 5 subfolders, with a total of 841 images; the 20 Rupee coin folder is distributed across 2 subfolders, containing a combined total of 324 images; the 25 Paisa coin folder is divided into 2 subfolders, housing 223 images in total; and the 50 Paisa coin folder comprises 4 subfolders, containing a cumulative total of 535 images. These are high quality images in .jpg format with 575 × 768 dimensions. The images were captured in different backgrounds, different lighting conditions, and different angles both from observe and reverse sides of the coin. The coins are segregated in proper folders. The main folder “Indian Coin dataset” consist subfolders for 1 Rupee Coin, 2 Rupee Coin, 5 Rupee Coin, 10 Rupee Coin, and 20 Rupee Coin, 50 Paisa Coin, 25 Paisa Coin namely. Inside each of these subfolder the coins are arranged in subfolder with the respective name of Indian motifs or symbols that are incorporated into the coin. The detail structure of the Indian coin directory and sub directory is shown in Fig. 1.

**Fig. 2:**
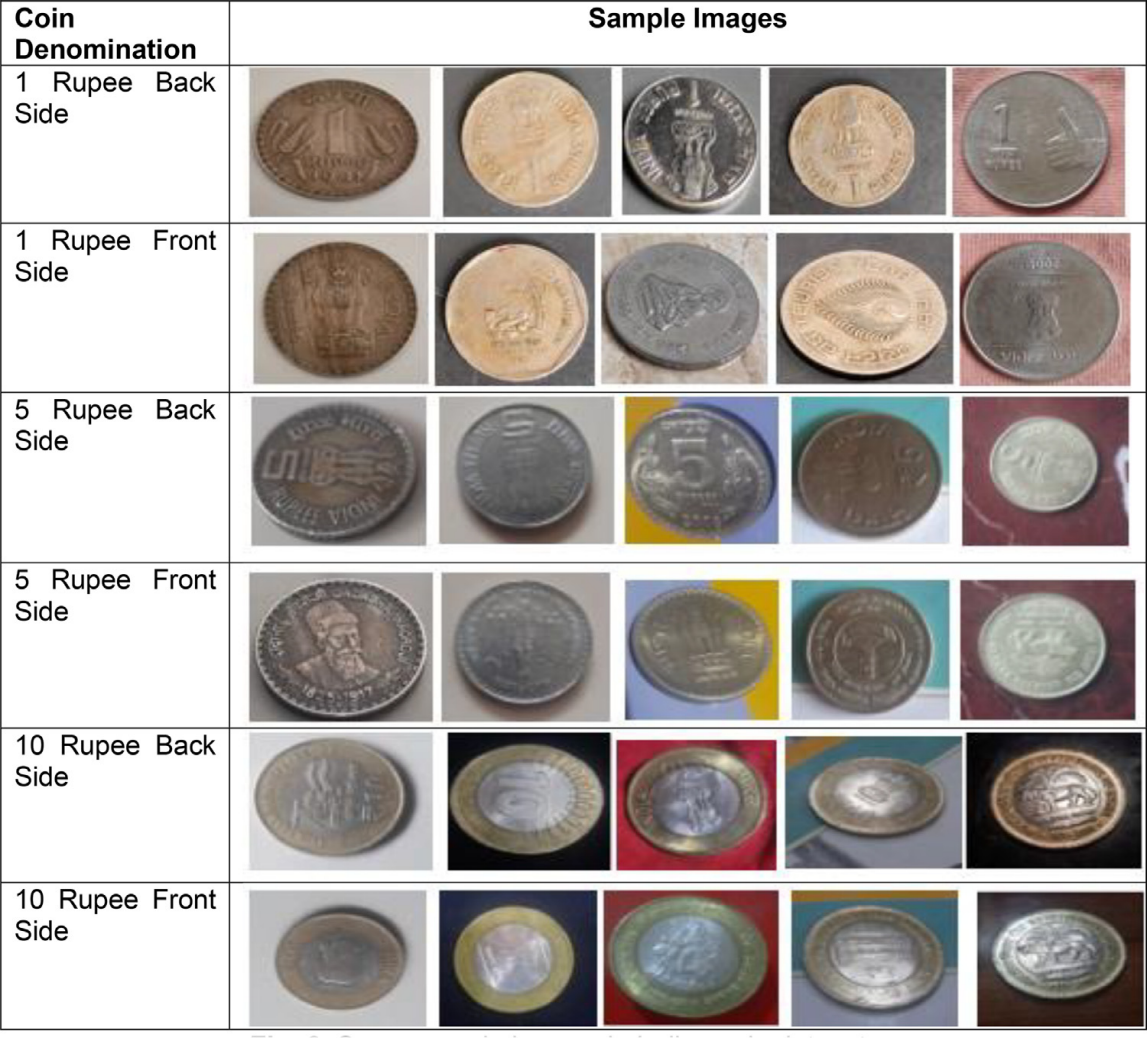
Some sample images in Indian coin dataset.

Subfolder “1 Rupees Coin” contains subfolders namely 8th World Tamil Conference, Cellular Jail, Embelem_1, Embelem_2, Embelem_3, Embelem_4, Food and Nutrition, Saint Dnyaneshwar, Tourism year. Subfolder “2 Rupees Coin” contains subfolder namely Agriexpo, Asian Games, Chatrapati Shivaji Maharaj, Deshbandhu Chitranjan Das, Dr. Syama Prasad Mookerjee, Flower and Emblem, Happy Family, Hastmudra, India Map, Louis Braille, Railways Elephant, Sant Tukaram, Shri Aurobindo, Supreme Court of India, World Food Day. Subfolder “5 Rupees Coin” contains subfolders namely 150 Anniversary of Motilal Nehru, 150 Birth Anniversary of Madan Mohan Malviya, 50th Anniversary of United Nations, C Subramaniam, Civil Aviation, Dadabhai Naoroji, Dandi March, Emblem, Govt Mint Kolkata, Indian Council of Medical, Mahatama Basaveshwar, Mother Teresa, Reserve Bank of India, Satyamev Jayate, Swami Vivekananda, World of work. Subfolder “10 Rupee Coin” contains subfolders namely 75th Year of Independence, Emblem, Mata Vaishno Devi, National Archives of India, Reserve Bank of India. Subfolder “20 Rupees Coin” contains subfolders namely 75th Year of Independence, Emblem. Subfolder “25 Paisa Coin” contains subfolders namely Emblem, Rhino. Subfolder “50 Paisa Coin” contains subfolders namely 50 Year of Independence, Emblem, Indira Gandhi, Map. Fig. 2 presents few sample images in dataset. As shown the images are captured in different backgrounds, lighting conditions, and angles, capturing both the obverse and reverse sides of the coin.

In the 1988 coin series, first time in India 10 paisa, 25 paisa, and 50 paisa coins were introduced in Stainless Steel. Coins starting in 1992, Rs.1 coins were minted in Steel, and Rs.2 and Rs.5 coins in Copper Nickel were introduced, leading to the gradual replacement of note issues of Rs.1, Rs.2, and Rs.5 denominations by 2004 with the launch of the Unity in Diversity series. The 2007 Hast Mudra coin series, issued by the RBI, featured stainless steel coins of 50 paisa, 1 rupee, and 2 rupee denominations, showcasing various Hasta Mudras (hand gestures in Indian Classical dance). The 2011 coin series, introduced by the RBI, covered denominations of 50 paisa, Rs.1, Rs.2, Rs.5, and Rs.10, with identical designs for 50 paisa, Rs.1, Rs.2, and Rs.5, and the Rs.10 coin continued to be issued in bimetallic formats. Additionally, the RBI issued various anniversary coins honouring various Indian politicians (C Subramaniam, Pandit Madan Mohan Malaviya, Motilal Nehru, Indira Gandhi, etc.), saints (Saint Dyaneshwar, Sant Tukaram, etc.), kings (Chhatrapati Shivaji Maharaj etc.), philosophers (Mahatama Basaveshwar, Swami Vivekananda) and social workers (Mother Teresa).

## 4 Experimental Design, Materials and Methods

### 4.1 Experimental design

Over ten years, one of the authors, driven by a passionate interest in numismatics, diligently collected a diverse array of coins currently in circulation in India. This extensive collection encompasses various denominations, including 25 Paisa Coin, 50 Paisa Coin, 1 Rupee Coin, 2 Rupee Coin, 5 Rupee Coin, 10 Rupee Coin, and 20 Rupee Coin. By drawing upon this decade-long endeavor, the dataset boasts a wide spectrum of coins, reflecting the currency in active use. These coins were carefully examined and cataloged to capture both their obverse and reverse sides, ensuring a holistic representation.

### 4.2 Material

The Indian coin dataset was created by acquiring the images using the high-resolution rear camera of a Xiaomi M2101K6P. The mobile cameras were used to capture images of coins from both the old and new categories. The captured images were resized to 575 × 768 pixels using IrfanView software.

### 4.3 Method

**Fig. 3:**
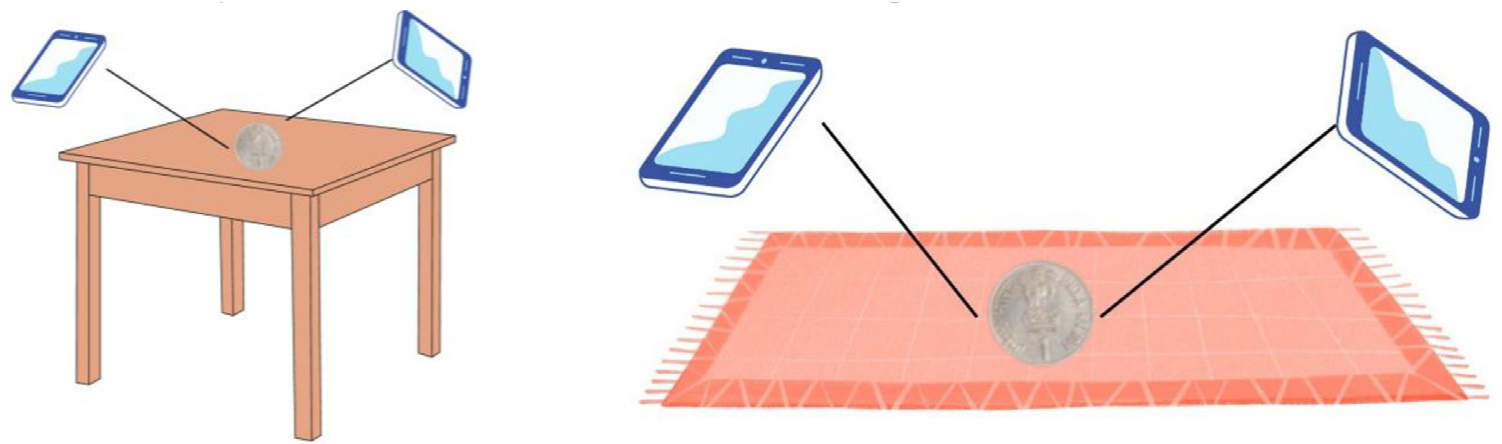
Illustrative representation of Indian coin image capturing method.

**Fig. 4:**
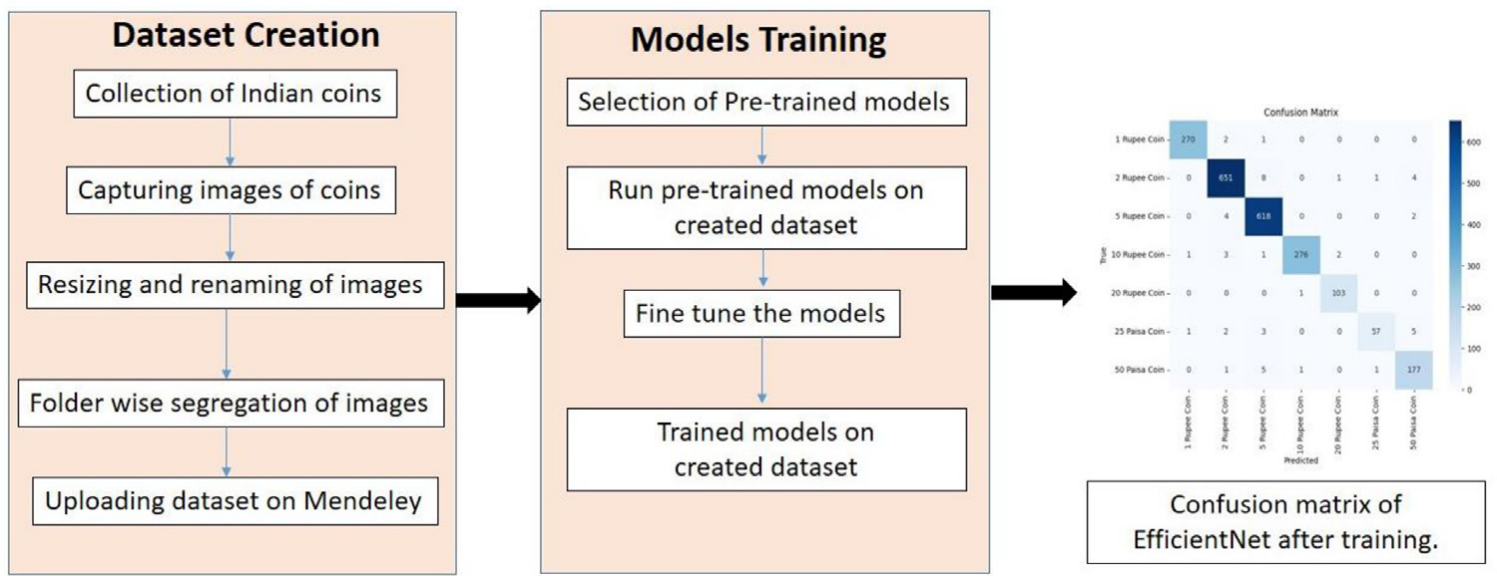
Stepwise methodology of dataset creation and model training.

The Indian coins were first collected, and then the coin images were captured using the rear camera of the mobile “Xiaomi M2101K6P”, from June to August 2023. The Coins were positioned against a range of backgrounds, and photographs were captured from various angles, including both sides (Fig. 3). After capturing the images they were resized to 575 × 768 size and segregated into respective folders as per the folder structure shown in Fig. 1. The detail steps for dataset creation are shown in Fig. 4.

The selected methodology for creating the (CIDCIC) stems from a recognition of the increasing digitalization of financial transactions and the corresponding need for robust tools to assist visually impaired individuals in managing their daily financial interactions. With this understanding, the dataset was curated to encompass a wide range of coin denominations and capture diverse visual characteristics, including shapes, sizes, and environmental contexts. The deliberate inclusion of various backgrounds, angles, and lighting conditions not only enriches the dataset but also addresses the limitations of existing datasets with a more extensive and up-to-date representation of contemporary Indian coins. These methodological choices aim to contribute significantly to the fields of computer vision, artificial intelligence, and machine learning by specifically addressing challenges related to coin detection, recognition, and the overall integrity of the monetary system. By enabling visually impaired individuals to independently identify and distinguish between different coin denominations, this dataset has the potential to enhance their participation in financial transactions and empower them in their daily lives. The resizing and organization of images further serve to standardize the dataset for analytical purposes, fostering transparency and collaboration within the research community.

Here are the specifics provided for camera specifications, background variations, data composition, image quality, and data accessibility.

#### 4.3.1 Camera specifications

Mobile Model: Xiaomi M2101K6P
Aperture: f/1.8
Exposure Time: 1/50 s.
ISO Speed: ISO-91
Focal Length: 5 mm
Metering Mode: Center-weighted average
Flash Usage: None

The Xiaomi M2101K6P mobile camera was selected for dataset creation due to its optimal specifications for coin photography. The f/1.8 aperture facilitates detailed imaging, crucial for capturing intricate coin features. With an exposure time of 1/50 s., a balance is struck between image sharpness and motion blur prevention. An ISO speed of ISO-91 minimizes digital noise while maintaining sensitivity to light. The 5 mm focal length allows for versatile framing without distortion. The center-weighted average metering mode ensures balanced exposure in various lighting conditions, while the deliberate avoidance of flash usage maintains the natural appearance of coins. These choices collectively contribute to a dataset that accurately represents Indian coins, catering to the needs of subsequent machine learning applications and analysis.

#### 4.3.2 Background variation

Dark
Illuminated
Different Angles
Obverse and Reverse Sides

#### 5.3.3 Dataset composition

Coin Denominations:
25 Paisa
50 Paisa
1 Rupee
2 Rupees
5 Rupees
10 Rupees
20 Rupees

#### 4.3.4 Image quality

Original images (6944 × 9280) pre-processed using IrafanView software.
Resized to 575 × 768 pixels.
Images saved in separate folders by denomination.

#### 4.3.5 Data accessibility

The Indian Coins Image Dataset is publicly accessible on Mendeley [12].

The timeline depicting the image collection and processing stages is provided in Table 1, whereas Table 2 display of respective image counts for each coin.

**Table 1:** Data acquisition steps.

Sr. No.	Step	Duration	Activity
1.	Image Acquisition	June to August	The images of coins from different angles, different backgrounds and environments were collected.
2.	Image Pre-processing	September to October	The images were resized and segregated in proper folders as per their denomination.

**Table 2:** Coin image collection details by denomination.

Sr. No	Directions & Environments Considered	Coins	Number of Images
1.	Observe & Reverse directions Dark & illuminated Background Various angles	1 Rupee	779
2.		2 Rupee	2042
3.		5 Rupee	1918
4.		10 Rupee	841
5.		20 Rupee	324
6.		25 Paisa	233
7.		50 Paisa	535
**Total**			**6672**

### 4.4 Demonstrating the significance of the dataset

In the landscape of machine learning datasets, recent advancements have yielded significant contributions tailored for machine learning applications. We aim to demonstrate the utility of the Indian coin image dataset through experiments conducted with established pre-trained models, namely EfficientNet, VGG16, and ResNet50. The primary objective is to evaluate the dataset's impact on enhancing the accuracy of machine learning models, particularly in the identification of Indian coins. Initially, the pre-trained models were applied without modifications, serving as a benchmark against our dataset. Subsequently, the models were fine-tuned using the Indian coin dataset, leading to a notable improvement in accuracy, particularly evident in the detection and classification of Indian coins (Fig. 4.).

**Fig. 5:**
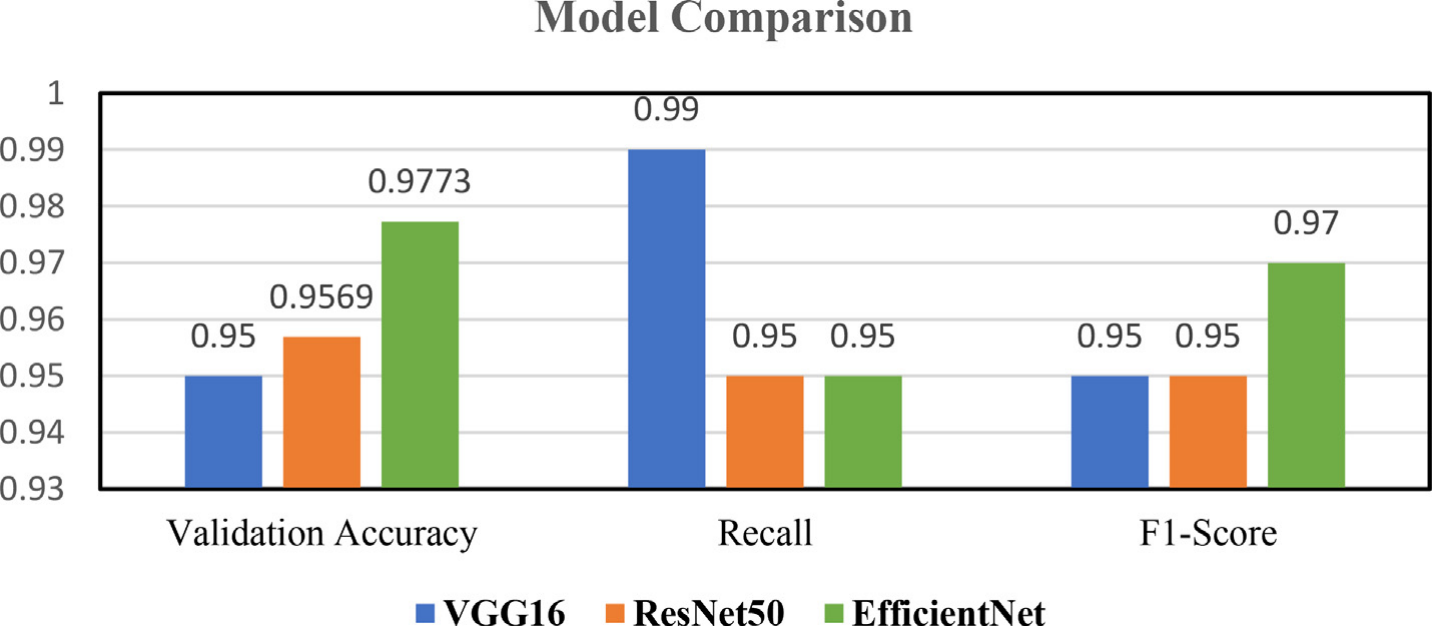
Comparative performance analysis of pre-trained models for coin image recognition.

**Fig. 6:**
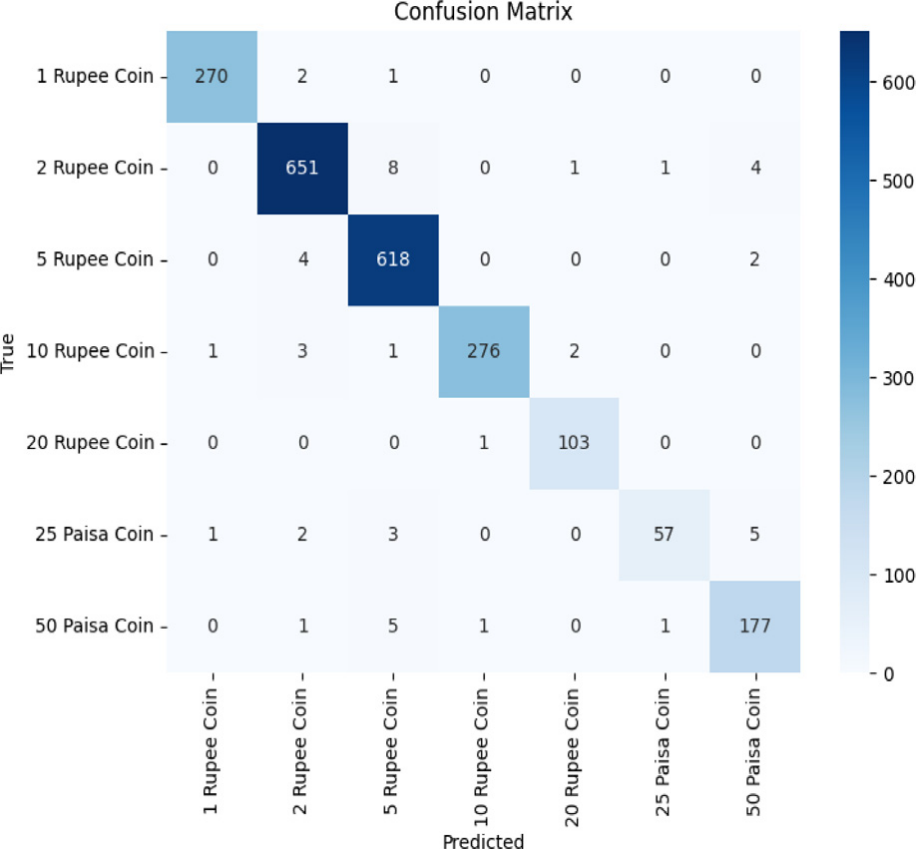
Confusion matrix of EfficientNet.

**Fig. 7:**
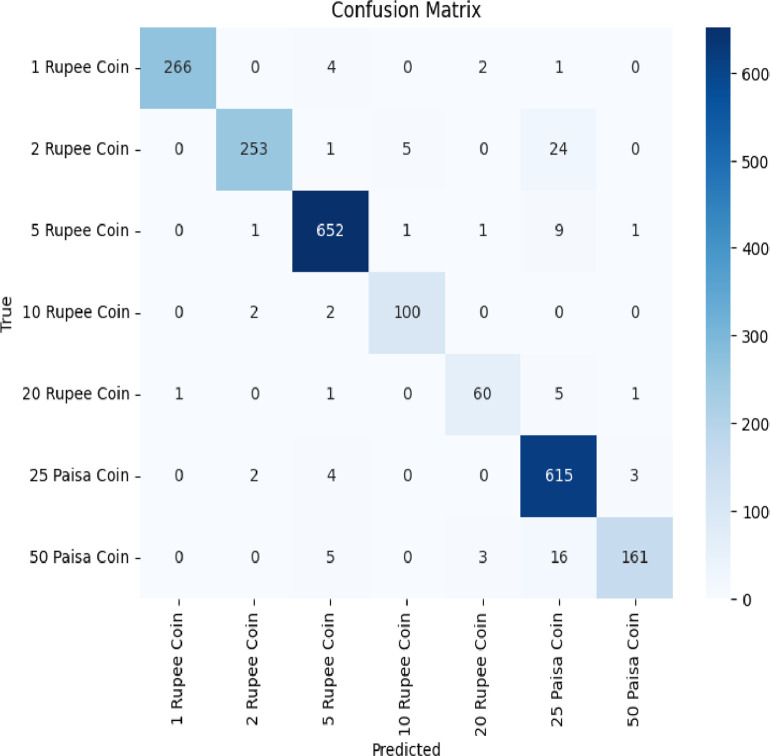
Confusion matrix of VGG16.

**Fig. 8:**
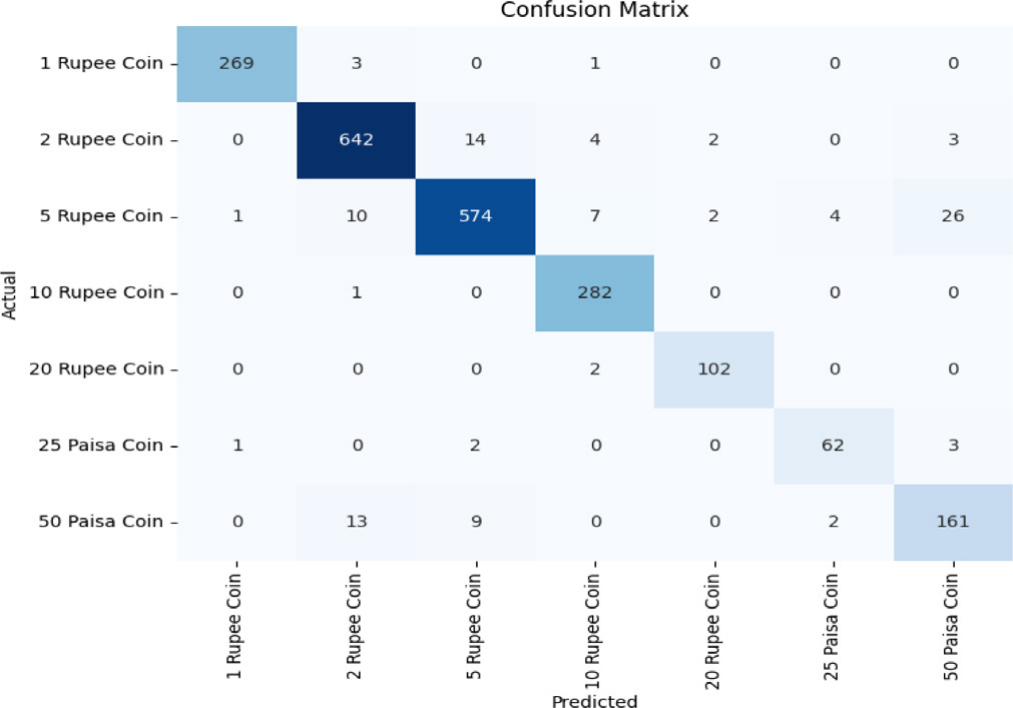
Confusion matrix of ResNet-50.

The pre-trained models are used to classify the Indian coin images as per their denomination. These pre-trained models namely EfficientNet, VGG16, and ResNet50 are chosen as they efficiently and accurately perform image classification after employing transfer learning to it. Accuracy, F1-score and Recall values of these three models are shown in Table 3 and plotted in Fig. 5.This Table 3 and Fig. 5 offers a comparative assessment of three pre-trained models, EfficientNet, VGG16, and ResNet50 - applied to the task of coin image recognition. Each model's performance is evaluated based on three critical metrics. EfficientNet exhibits an impressive validation accuracy of 97.73%, demonstrating precision in classifying coin images. VGG16, though slightly lower at 95%, excels in recall with an impressive rate of 99%, signifying high sensitivity to true positives. ResNet50 closely aligns with EfficientNet, achieving a validation accuracy of 95.69% and a recall rate of 95%. In terms of F1-Score, EfficientNet strikes a harmonious balance between precision and recall at 97%. VGG16 and ResNet50 both achieve an F1-Score of 95%, signifying reliable overall performance. This comparative analysis provides invaluable insights into the strengths of each model, aiding in the selection of the most suitable one for coin image recognition applications. The overall classification accuracy of EfficientNet, VGG16 and ResNet-50 is represented using the confusion matrix shown in Fig. 6, Fig. 7, Fig. 8 respectively. In the future, we plan to conduct a thorough investigation involving a range of cutting-edge deep learning models using this dataset. This analysis aims to identify the most effective technique for real-world applications.

**Table 3:** Performance metrics of pre-trained models for coin image recognition.

Pre-trained model	Validation accuracy	Recall	F1-Score
EfficientNet	0.9773	0.95	0.97
VGG16	0.9500	0.99	0.95
ResNet50	0.9569	0.95	0.95

In summary, our Indian coin image dataset proves instrumental in enhancing the performance of machine learning models, such as EfficientNet, VGG16, and ResNet50, by serving as a robust resource for training and fine-tuning, consequently contributing to the more accurate identification of Indian coins.

### 4.5 Potential applications of dataset

The Indian Coin Image Dataset presents a versatile tool with a multitude of practical applications. It holds the potential to revolutionize accessibility for visually impaired individuals, enabling them to independently identify and differentiate coin denominations in daily financial transactions. Moreover, industries involving currency management, such as banks and retail, stand to benefit from enhanced coin recognition systems, potentially streamlining processes like sorting and counting. Numismatic enthusiasts and researchers can tap into the dataset's wealth of coin images for detailed studies on historical and monetary significance. Additionally, this dataset could find application in education, online auctions, anti-counterfeiting measures, and museum exhibits, impacting a wide array of sectors from finance to cultural preservation. Its adaptability and impact across diverse fields make the Indian Coin Image Dataset a valuable resource with far-reaching potential.

## 5 Conclusion

Indian Coins (CIDCIC) marks a significant stride in addressing the growing need for expansive and diverse datasets in the digital age. The dataset, comprising 6672 images encompassing 53 different classes of Indian coins, offers a rich and multifaceted resource for researchers, practitioners, and technology developers in the realms of computer vision, artificial intelligence, and machine learning. The key findings of this research underscore the dataset's comprehensive nature, including images from various angles, environments, backgrounds, and directions. This diversity not only enhances its representativeness but also contributes substantially to overcoming the limitations observed in existing datasets. By encompassing denominations ranging from 25 Paisa to 20 Rupee, the CIDCIC provides a holistic understanding of contemporary Indian coins, addressing the need for a broader and more up-to-date representation. The dataset's potential to aid in coin detection and recognition holds immense value in enhancing the independence of visually impaired individuals during their daily financial transactions. Future research endeavors could explore advanced techniques within the domains of computer vision and machine learning to refine and optimize coin detection and recognition algorithms further.

## Limitations

The dataset is comprehensive collection of Indian coins; thus, it potentially limits its applicability to classification and recognition if Indian coins only. At the time of writing this article, the dataset does not include the coins that have been discontinued from circulation. The absence of discontinued coins from circulation raises a limitation in reflecting the entirety of India's numismatic history, impacting the dataset's comprehensiveness. The dataset does not encompass the entire spectrum of diverse coins periodically issued by the Reserve Bank of India.

## CRediT authorship contribution statement

**Yogesh Suryawanshi:** Conceptualization, Methodology, Validation, Writing – original draft. **Vidula Meshram:** Writing – original draft. **Kailas Patil:** Conceptualization, Supervision, Writing – review & editing. **Michael Testani:** Writing – review & editing. **Prawit Chumchu:** Writing – review & editing. **Anmol Sharma:** Methodology.

## Data Availability

Image Dataset of Indian Coin (Original data) (Mendeley Data)
